# Cyto-nuclear discordance in the phylogeny of *Ficus *section *Galoglychia *and host shifts in plant-pollinator associations

**DOI:** 10.1186/1471-2148-9-248

**Published:** 2009-10-12

**Authors:** Julien P Renoult, Finn Kjellberg, Cinderella Grout, Sylvain Santoni, Bouchaïb Khadari

**Affiliations:** 1 CNRS, UMR 5175 Centre d'Ecologie Fonctionnelle et Evolutive (CEFE), Equipe Interactions Biotiques, F-34293 Montpellier Cedex 5, France; 2 INRA, UMR 1098, Développement et Amélioration des Plantes (DAP), Campus CIRAD TA A 96/03, Av. Agropolis, 34398 Montpellier Cedex 5, France; 3 Montpellier SupAgro, UMR 1098, Développement et Amélioration des Plantes (DAP), Bat. 2, Campus CIRAD TA A 96/03, Av. Agropolis, 34398 Montpellier Cedex 5, France; 4 INRA, UMR 1097, Diversité et Adaptation des Plantes Cultivées (DiA-PC), Bat. 33, 2 place Viala, 34060 Montpellier Cedex 2, France; 5 Conservatoire Botanique National Méditerranéen de Porquerolles, UMR 1098, 76 A, Av. Gambetta, 83400 Hyères, France

## Abstract

**Background:**

Hybridization events are relatively common in vascular plants. However, the frequency of these events is unevenly distributed across the plant phylogeny. Plant families in which individual species are pollinated by specific pollinator species are predicted to be less prone to hybridization than other families. However, exceptions may occur within these families, when pollinators shift host-plant species. Indeed, host shifts are expected to increase the rate of hybridization events. Pollinators of *Ficus *section *Galoglychia *are suspected to have changed host repeatedly, based on several cases of incongruence between plant phylogeny and taxonomy, and insect phylogeny and taxonomy. We tracked cyto-nuclear discordance across section *Galoglychia *as evidence for hybridization. To achieve a proper global view, we first clarified the monophyly of section *Galoglychia *as it had been questioned by recent phylogenetic studies. Moreover, we investigated if fig size could be a factor facilitating host shifts.

**Results:**

Phylogenetic chloroplast and nuclear results demonstrated the monophyly of section *Galoglychia*. Within section *Galoglychia*, we detected several cases of statistically significant cyto-nuclear discordance. Discordances concern both terminal nodes of the phylogenetic trees and one deep node defining relationships between subsections. Because nuclear phylogeny is congruent with morphological taxonomy, discordances were caused by the chloroplast phylogeny. Introgressive hybridization was the most likely explanation for these discordances. We also detected that subsections pollinated by several wasp genera had smaller figs and were pollinated by smaller wasps than subsections pollinated by a single wasp genus.

**Conclusion:**

As hypothesized, we discovered evidences of past hybridization in *Ficus *section *Galoglychia*. Further, introgression was only detected in subsections presenting incongruence between plant and pollinator phylogenies and taxonomy. This supports the hypothesis that host shift is the cause for plant-pollinator incongruence. Moreover, small fig size could facilitate host shifts. Eventually, this study demonstrates that non-coding chloroplast markers are valuable to resolve deep nodes in *Ficus *phylogeny.

## Background

Frequent natural hybridization events in vascular plants were documented by early biologists (e.g. [1,2]). However, the prevalence of hybridization events is unevenly distributed across the plant phylogeny and seems to be concentrated within a small fraction of families and genera [3]. Grant [4] pointed out that the frequency of natural hybridizations varies with factors such as life history, breeding system, environmental disturbance, genetic predisposition, and eventually pollination syndrome. In this context, plant species pollinated by specific pollinator species are predicted to present almost no hybridization events. Indeed, in specific mutualisms as plant-pollinator associations, the associated species usually present co-adaptations involved in attraction, recognition and physical compatibility with the other species. Such adaptations can be viewed as pre-zygotic barriers limiting interspecific hybridization [5-7].

Host shift is one of the main ecological processes that can break patterns of strict cospeciation between tightly bound interacting species [8-11], as observed in some cases affecting postulated strict co-evolutionary pattern in specific pollination mutualisms [12]. When associated with a transitional use of two hosts by the pollinator, a host shift may allow plant hybridization and hence leave a signature of genetic introgression in the plant genome. Following such hybridization, recurrent backcrosses of offspring with one parent may also allow the introgression of a limited set of foreign alleles into a plant species [13]. Hence, plant lineages in which pollinators have jumped from one host to another are more likely to exhibit signs of introgression than other lineages.

Detecting past events of host shifts requires tracking incongruence between phylogenetic trees of interacting taxa [8,14,15]. However, pollinator species duplication in the absence of host speciation, followed by cospeciation and subsequent asymmetric extinction, a phenomenon we will call duplication/extinction hereafter, is another mechanism that can cause incongruence between host phylogeny and partner phylogeny [16]. Although different in essence, these phenomena lead to similar patterns of phylogenetic incongruence, and discriminating between them is often largely speculative [17]. Nevertheless, and contrary to host shift, duplication/extinction does not involve plant hybridization. Consequently, detecting introgressed genes in a plant lineage for which there is incongruence between plant and pollinator phylogenies would support the host shift hypothesis.

Fig trees (*Ficus*, Moraceae) and their pollinating wasps (Agaonidae, Chalcidoidae) constitute one of the well-known cases of species-specific pollination mutualism. In this system, the rule is that a wasp species generally pollinates a single host species [18,19]. Further *Ficus *and Agaonid wasp morphological taxonomy and molecular phylogenies are steadily improving, providing a unique set of information to infer past history of the association on a broad set of species. Based on phylogenetic data, several authors have suggested parallel diversification of host and associated pollinating wasps [5,20-23]. Moreover, fig tree sections or subsections appear to be generally monophyletic and pollinated by a single wasp genus or by a few genera. Nevertheless, exceptions to the rule of a single agaonid wasp species associated with a single *Ficus *species are quite frequent and rather well documented based on morphology [24,25] as well as on molecular methods [12,26-28]. Indeed, Rasplus *et al. *[29] estimated for Africa, on the basis of morphology, that 17% of fig species were pollinated by more than one wasp species and that 15% of fig pollinator wasps used two or more hosts. However, molecular data on Australian pollinators suggest that when two or more wasps pollinate the same host they seem to often be sister species [30].

**Figure 1:**
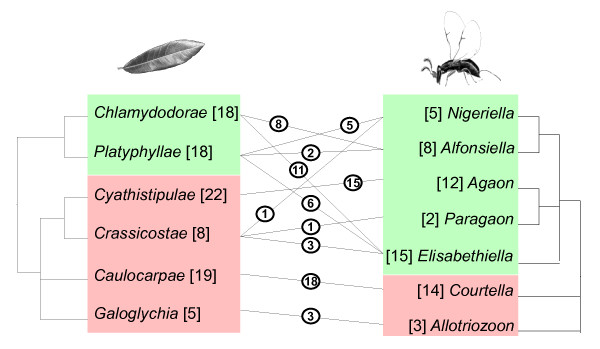
**Association between subsections of section *Galoglychia *in *Ficus *(left) and their genera of Agaonidae pollinating wasps (right)**. Numbers of taxa described for each wasp genus and each fig subsection are given within brackets. Linking lines inform on pollination associations between wasp genera and host subsections, with, for each link, the number of fig species known to be pollinated by a given wasp genus provided within circles. Colours highlight major clades to stress the discrepancy between plant (from [33]) and wasp (from [26]) phylogenies.

Within this general context, species of section *Galoglychia *show an unusual pattern of association with their pollinating wasps within genus *Ficus*. The 77 described species of section *Galoglychia *are distributed into six subsections [18,31], several of which are unambiguously defined. Seven morphologically unambiguous genera of fig wasps are known to pollinate section *Galoglychia *(Figure 1). Some genera are both subsection specific and the sole pollinators of the subsection. Other genera pollinate fig trees from different subsections and some subsections are pollinated by several wasp genera. Further some individual *Ficus *species may be pollinated by wasps belonging to different genera (e.g. *Ficus natalensis *pollinated by *Alfonsiella *and *Elisabethiella*) and one wasp species may pollinate several *Ficus *species (e.g. *Elisabethiella stuckenbergii *pollinates *F. burkei, F. natalensis, F. petersii and F. lingua depauperata *[32]).

Recent molecular phylogenies supported, with some caveats, the monophyly of subsections within section *Galoglychia *[33] and that of wasp genera [26]. Hence the interactions between figs of section *Galoglychia *and their pollinating wasps present us with a series of instances of lack of strict specificity, and lack of congruence between fine wasp and *Ficus *taxonomy and phylogeny [18,34]. Comparing the phylogenies of trees and insects suggests additional lack of congruence, with respect to the order of branching of *Ficus *subsections and wasp genera (Figure 1). In the plant phylogeny [33], based on ITS and ETS nuclear markers, subsections *Cyathistipulae *and *Crassicostae *are grouped with subsections *Galoglychia *and *Caulocarpae *within a clade, while the remaining subsections are clustered together into a second clade. In the wasp pollinator phylogeny [26], based on 28S and ITS markers, subsection *Galoglychia *pollinators and *Caulocarpae *pollinators constitute the outgroups of a clade including the pollinators of the remaining subsections. Fig tree phylogeny is thus incongruent with pollinating wasp phylogeny with respect to the branching of subsections *Cyathistipulae *and *Crassicostae *versus *Chlamydodorae *and their pollinators. An additional finding of the molecular phylogeny of section *Galoglychia *is a suggestion that neotropical species of section *Americana *could be enclosed within section *Galoglychia*, although this result was only weakly supported by the various node validation methods [33]. If section *Galoglychia *is really paraphyletic to section *Americana*, pollinators of this last section could provide an example, pending phylogenetic and taxonomic re-evaluation, of discrepancy between the phylogenetic histories of *Ficus *and their pollinating wasps as the pollinators of section *Americana *and of section *Galoglychia *have been classified into different subfamilies (Agaoninae and Blastophaginae) [19].

Incongruence between pollinator and host phylogenies has been traditionally attributed to host shifts [12,35]. However, ancient pollinator species duplication events could also have lead to different wasp pollinator genera pollinating the same host figs. Subsequent cospeciation and asymmetric extinction could be responsible of the apparently haphazard host association exhibited by *Alfonsiella *and *Elisabethiella *pollinators [26,36].

The goal of this contribution is to study introgression events in the phylogeny of section *Galoglychia*. If host shifts and not duplications/extinctions are effectively responsible for plant-pollinator taxonomic and phylogenetic incongruence, then we expect to detect introgression events, especially in the plant lineages implied in this incongruence. We compared nuclear and chloroplast phylogenies to detect cases of genetic introgression between *Ficus *species, because the chloroplast genome of vascular plants possesses several features that facilitate its introgression into a new plant species after hybridization compared to the nuclear genome [13,37,38], as illustrated by the numerous cases of chloroplast introgression documented in the literature [37,39,40]. Our working hypothesis is that the current complex pattern of host association of genera *Elisabethiella*, *Nigeriella *and *Alfonsiella *result from host shifts. The prediction is that we may detect evidence for cytoplasm transfer between species of subsection *Chlamydodorae*, *Platyphyllae *and *Crassicostae*. We will also search for other potential cases of genetic introgression and especially for subsection *Cyathistipulae *for which available data suggests mismatch between the phylogenetic position of the subsection [33] and the phylogenetic position of its pollinators, namely genus *Agaon *[26]. To do so, we first developed specific chloroplast markers for *Ficus *and sequenced them in 58 individuals representing 38 species of section *Galoglychia *and 10 species of section *Americana*. We also sequenced ITS and ETS markers on these same individuals. Second, we searched for cases of discordance between chloroplast and nuclear phylogenies and tested their significance. Third, we showed that genetic introgression was the most likely mechanism to explain the cases of discordance. Besides, because section *Galoglychia *is the sole *Ficus *section subdivided into six subsections all of which are well defined (pending revision of the taxonomic position of some species assigned to subsection *Platyphyllae*, see discussion) and the sole section pollinated by six monophyletic agaonid wasp genera, this section offers a unique opportunity to investigate host shifts and their determinants. Within the section, known and suspected examples of pollinators using several hosts seem to involve host species presenting rather small figs, such as *F. burkei, F. natalensis, F. petersii *and *F. lingua depauperata *[32]. Therefore we investigated whether the fig subsections involved effectively presented small figs and whether their pollinator genera presented small body size.

## Results

### Data set partitioning

Chloroplast marker development allowed producing fourteen pairs of primer specifically developed for *Ficus *(see Additional file 1). Five of them - *atpB-rbcL, FcB, FcJ, FcL *and *trnL-trnF *- were selected for this analysis based on the phylogenetic information provided by a subsample of the final data set used in this study. The final combined chloroplast matrix contained 58 accessions and 3,604 base pairs (see Additional file 2; TreeBASE accession number: SN4278). One hundred and twenty-two sites were variable and 52 (1.5%) were potentially parsimony informative. The model of sequence evolution varied across chloroplast partitions (Table 1). We used arithmetic mean -lnL of Bayesian posterior topologies to measure the ability of data partitioning to explain the entire data set. The analysis separating non-coding and coding regions (strategy *S*_3_) of the chloroplast data set returned a decisively better description of the data than the other two analyses according to Bayes factors (Table 2).

**Table 1:** Data partitions, genome to which they are associated, total number of characters of each partition used in phylogenetic analyses and their estimated model of sequence evolution.

**Partition**	**Genome**	**Number of characters**	**Selected Model**
*P*_1_: All chloroplast data combined	Chloropast	3604	GTR+I+Γ
*P*_2_: *atpB-rbcL*	Chloropast	820	HKY+Γ
*P*_3_: *FcB*	Chloropast	779	GTR
*P*_4_: *FcJ*	Chloropast	760	HKY
*P*_5_: *FcL*	Chloropast	777	GTR+Γ
*P*_6_: *trnL-trnF*	Chloropast	468	HKY
*P*_7_: Coding chloroplast DNA	Chloropast	964	HKY
*P*_8_: Non-coding chloroplast DNA	Chloropast	2640	GTR+Γ
*P*_9_: All nuclear data combined	Nuclear	1320	GTR+Γ
*P*_10_: *ITS*	Nuclear	810	GTR+Γ
*P*_11_: *ETS*	Nuclear	510	HKY+Γ

**Table 2:** Arithmetic mean -LnL for each partition strategy (*S*_*i*_) and 2ln Bayes factors results of comparisons between the strategies with the highest likelihood and alternative strategies.

**Partition strategy**	**Mean -lnL**	**2ln Bayes factor**
*S*_1_: *P*_1_	6158.25	*S*_1 _vs *S*_3_: 214.3
*S*_2_: *P*_2_*+P*_3_*+P*_4_*+P*_5_*+P*_6_	6030.59	*S*_2 _vs *S*_3_: 11.38
*S*_3_: *P*_7_*+P*_8_	6028.45	
*S*_4_: *P*_9_	5584.63	*S*_4 _vs *S*_5_: 21.06
*S*_5_: *P*_10_*+P*_11_	5578.51	

The combined ITS and ETS matrix contained 1,320 base pairs of which 348 were variable and 158 (12%) were potentially parsimony informative. The model of sequence evolution differed between the ITS and ETS partitions (Table 1). The strategy individualizing each of these partitions (*S*_5_) was decisively better than the strategy combining the entire nuclear data set (*S*_4_) (Table 2). In the following we will only present the results obtained using the best strategy for each data set.

### Chloroplast phylogeny of section *Galoglychia*

**Figure 2:**
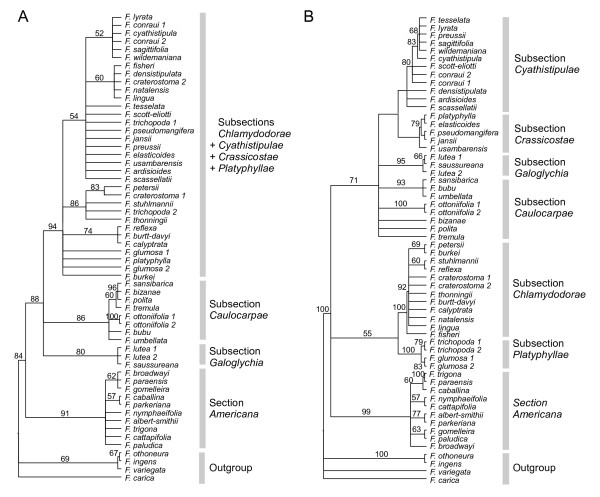
**Strict consensus trees of all most parsimonious trees**. Analyses performed with (A) the chloroplast data set (length = 141 steps) and (B) the nuclear ITS+ETS data set (length = 521 steps). Values above branches indicate bootstrap supports.

**Figure 3:**
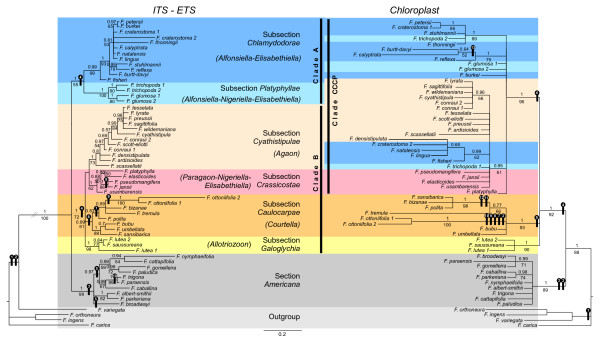
**ITS+ETS (left) and chloroplast (right) phylogenetic trees obtained with the Bayesian Inference (BI) analyses**. Values above and under branches indicate posterior probability of BI and bootstrap support performed with the Maximum Likelihood analysis, respectively. Synapomorphic indels of each data set are mapped and numbered onto the corresponding tree (details about indels are given in Additional file 3). Genera of wasps known to pollinate a subsection are indicated between brackets.

The combined chloroplast matrix produced twelve most parsimonious trees of length 141. Consistency and Retention Indexes (CI = 0.88; RI = 0.93) suggested the presence of a single island of trees [41]. The strict consensus of all most parsimonious trees (SCMP) is shown in Figure 2A with bootstrap percentages (MP_BS). The two different runs during the BI analysis with partitioning strategy *S*_3 _reached model parameter convergence and generated a similar tree. This tree is shown in Figure 3 with the posterior probabilities (PP) indicated for each node. ML analysis produced one most likely tree (-lnL = 5960.04; not shown), slightly less well resolved than the Bayesian tree. Bootstrap percentages (ML_BS) are indicated over the Bayesian tree in Figure 3.

The SCMP tree, the ML and the Bayesian trees were all similar in their overall topology. The monophyly of section *Americana *was strongly supported (91;89;1 for %MP_BS, %ML_BS and PP respectively; node supports are given in a similar order in the following) and formed a sister clade to section *Galoglychia *which was monophyletic (88;92;1). Subsections *Galoglychia *and *Caulocarpae *constituted monophyletic groups (80;90;1 and 86;93;1, respectively) and were basal within section *Galoglychia*. A third well supported clade was composed by species belonging to subsections *Cyathistipulae*, *Crassicostae*, *Chlamydodorae *and *Platyphyllae *(94;96;1; CCCP clade hereafter). However and contrary to the previous subsections, clade subsections did not form monophyletic groups within the CCCP. For example, the two accessions of *F. trichopoda *branched within distinct clades, as did the two accessions of *F. craterostoma*, and *F. densistipulata *(subsection *Cyathistipulae*) branched within a clade only composed of taxa of subsection *Chlamydodorae*.

One hundred and four distinct gaps were coded for the chloroplast data set. Five insertions and eight deletions were synapomorphic (Figure 3; see Additional file 3). A 10 bp insertion (chloroplast indel n°4) supported the monophyly of section *Galoglychia *since it was found neither in the outgroup nor in section *Americana*. A 7 bp deletion (n°6) supported the CCCP clade.

### Nuclear phylogeny of section *Galoglychia*

The combined nuclear matrix produced 15,000 most parsimony informative trees of length 521 (RI = 0.84; CI = 0.75). The SCMP of all 15,000 trees is shown in Figure 2B. Figure 3 illustrates the unique Bayesian tree produced by the two different runs during the analysis and using the *S*_5 _partitioning strategy. Only one most likely tree (-lnL = 5150.78; not shown) was produced by the ML analysis. As with the chloroplast data, it differed from the Bayesian tree only in being slightly less resolved.

Here again, the SCMP, the ML and the Bayesian trees were all similar in their overall topology. A basal trichotomy divided the ingroup into three clades. The first one was the strongly supported section *Americana *(91;99;1). The second grouped subsection *Chlamydodorae *and part of subsection *Platyphyllae *(Clade A). Although it was not strongly supported by bootstraps (55;55), this clade was found in the 15,000 most parsimonious trees, and had a Bayesian posterior probability of one. The third clade grouped accessions from subsections *Caulocarpae*, *Galoglychia*, *Cyathistipulae*, *Crassicostae *and part of *Platyphyllae *(Clade B). It was not strongly supported by the bootstrap distribution (71;72) but it was present in every most parsimonious trees and is maximally supported by the BI analysis.

Sixty five indels were coded for the nuclear data set and 10 of them were synapomorphic (Figure 3; see Additional file 3). The ingroup is supported by one deletion and one insertion. Inside the ingroup, all accessions of section *Americana *presented a 3 bp deletion in their ETS sequence while all accessions of section *Galoglychia *shared a one base pair insertion in the ITS sequence.

### Investigation of the cyto-nuclear discordance

While different phylogenetic reconstruction methods gave rise to similar topologies for a given data set, chloroplast and nuclear phylogenies appeared discordant for several internal and external nodes. Templeton tests applied to MP trees, and SH tests to ML trees always returned congruent results (Table 3). Both SCMP and ML trees explained significantly better the data set used to reconstruct these trees than the rival data set, demonstrating the overall discordance between nuclear and chloroplast topologies. We then tested the significance of local discordances involving *F. trichopoda*, *F. craterostoma *and *F. densistipulata *accessions. The original chloroplast topology in which both accessions of *F. trichopoda *are paraphyletic explained significantly better the chloroplast data than a modified topology in which these accessions were branched to form a monophyletic relationship. The same was true with the *F. craterostoma *chloroplast accessions. However, the original chloroplast topology was not significantly better than the topology in which *F. densistipulata *was branched with other *Cyathistipulae *taxa to make this subsection monophyletic. Alternatively, modifying the nuclear topology to branch one of the two *F. trichopoda *and one of the two *F. craterostoma *accessions with *Chlamydodorae *and with *Cyathistipulae *and *Crassicostae *accessions respectively, as in the chloroplast topology, produced significantly worse topologies than the unmodified nuclear topologies for the nuclear dataset. The same was true when *F. densistipulata *was branched with the *Chlamydodorae *species as in the chloroplast topology. For these last tests, it was possible to artificially reconstruct several alternative topologies. Table 3 gives results only for possibilities giving the highest *P*-value.

**Table 3:** Results of Templeton tests with most parsimonious (MP) trees and SH tests with maximum likelihood (ML) trees.

	**Templeton test**		**SH test**	
	
**Topology**	**Length**	***P*-Value**	**-LnL**	***P*-Value**
*Chloroplast Data*				
**Chloroplast**	**143**		**5956.50**	
Nuclear	195	< 0.001	6241.39	< 0.001
chloroplast - *F. trichopoda *monophyletic	147	0.045	5981.30	0.021
chloroplast - *F. craterostoma *monophyletic	152	0.003	5999.10	0.001
chloroplast - *F. densistipulata *within *Cyathistipulae*	144	0.564	5961.60	0.280
				
**(*Americana*, (*Galoglychia, Caulocarpae*,(*Cyathistipulae *+ *Crassicostae *+ *Chlamydodorae *+ *Platyphyllae*))**	**226**		**6061.80**	
(*Americana*, (*Galoglychia *+ *Caulocarpae *+ *Cyathistipulae *+ *Crassicostae*), (*Chlamydodorae *+ *Platyphyllae*))	276	0.016	6340.26	0.005
				
*Nuclear Data*				
**Nuclear**	**539**		**5170.68**	
Chloroplast	741	< 0.001	6091.60	< 0.001
nuclear - *F. trichopoda *2 within *Chlamydodorae*	560	< 0.001	5259.70	0.000
nuclear - *F. craterostoma *2 within *Cyathistipulae *and *Crassicostae*	560	< 0.001	5211.23	0.001
nuclear - *F. densistipulata *within *Chlamydodorae*	559	< 0.001	5256.40	< 0.001
				
**(*Americana*, (*Galoglychia *+ *Caulocarpae *+ *Cyathistipulae *+ *Crassicostae*), (*Chlamydodorae *+ *Platyphyllae*))**	**818**		**5407.92**	
(*Americana*, (*Galoglychia, Caulocarpae*, (*Cyathistipulae *+ *Crassicostae *+ *Chlamydodorae *+ *Platyphyllae*))	962	0.001	5890.20	0.002

*P*-values of a given line correspond to the test comparing the length (MP trees) or the likelihood (ML trees) of the topology of the corresponding line with the length/likelihood of the above topology written in bold characters. In the topology description, "," indicates that branching between subsections are left unresolved, and "+" indicates that the branching among and between subsections are left unresolved. Likelihoods were computed with PAUP.

Besides the terminal cases of discordance tested above, a cyto-nuclear discordance was generated by the branching of subsections *Cyathistipulae *and *Crassicostae*. To avoid interference due to cases of terminal discordance, we tested the significance of this deep discordance by comparing topologies differing only by the branching of these two subsections (Table 3). Therefore, branching was left unresolved (i.e. with a polytomy) inside subsections *Galoglychia *and *Caulocarpae *for the chloroplast topology, and inside the CCCP clade, Clade A and Clade B (see Figure 3). Both Templeton and SH tests showed that the position of subsections *Cyathistipulae *and *Crassicostae *obtained in trees reconstructed using one data set (nuclear or cytoplasmic) produced a significantly better topology than the rival topology for that set of data.

The Bayesian approach used to statistically evaluate discordances gave similar results (not shown). For each Bayesian analysis, we did not find any branching supporting a rival phylogenetic hypothesis in any tree of the 95% credible set sampled once stationarity was reached. The sole exception was constituted by *F. densistipulata *in the chloroplast data set analysis, which branched with other *Cyatistipulae *species in 17% of the trees of the 95% credible set.

**Figure 4:**
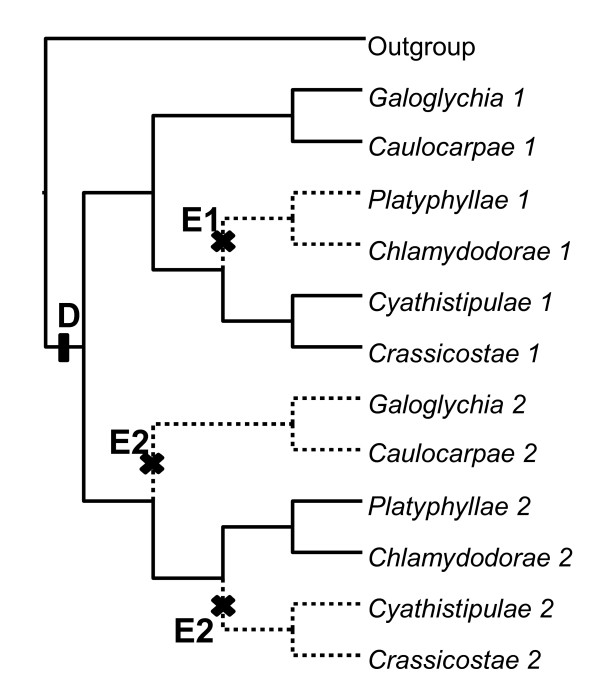
**The cyto-nuclear discordance explained by ITS and ETS duplication-extinction**. The scenario requires a basal duplication (D) followed by three extinction events: one extinction of allele 1 (E1) and two extinctions of allele 2 (E2).

To check that apparent cyto-nuclear incongruence was not an artefact due to saturation by mutations, we plotted the estimated number of transitions and transversions against the TN93 [42] genetic distances (see Additional file 4). For all three analyses, transitions outnumber transversions indicating that substitutions are not saturated in the three types of data (chloroplast, ETS and ITS). Finally, considering ITS plus ETS as a single nuclear unit, a scenario based on gene duplication without genetic introgression and explaining the deep node discordance between nuclear and cytoplasmic phylogenies requires one duplication event at the base of section *Galoglychia *followed by three extinction events, each at the base of a group of subsections (Figure 4).

### Fig and pollinator size

**Figure 5:**
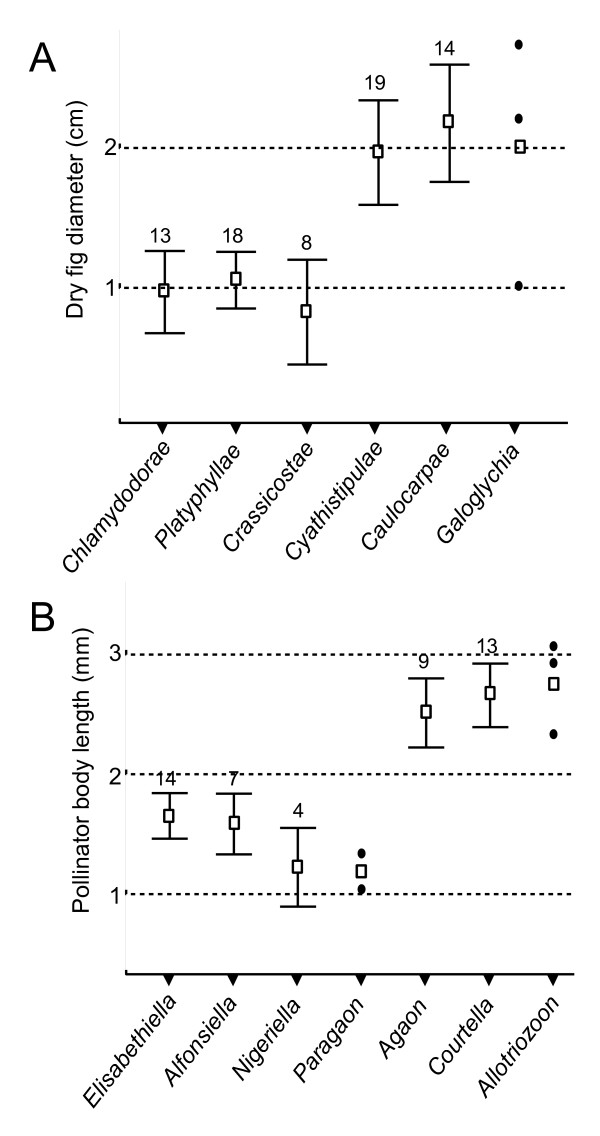
**Mean dry fig diameter and mean pollinator body length for each subsection of section *Galoglychia *and each pollinating wasp genus**. Figures above whiskers give the number (n) of species included in the mean calculation. Whiskers give 95% credible interval (provided when n>3, otherwise single values are plotted).

We investigated if fig and pollinator size were morphological traits associated at the subsection/genus level with plant-pollinator phylogenetic incongruence. Indeed, because of the as yet limited number of documented cases of incongruence, we limited our investigation to crude comparisons. Dry fig diameter of species belonging to section *Galoglychia *was distributed into two size categories (Figure 5A). Species belonging to subsections pollinated by a single pollinator genus (subsections *Cyathistipulae, Caulocarpae *and *Galoglychia*) presented significantly larger figs than species belonging to sections pollinated by at least two pollinator genera (*t *= 7.63; *p *< 10^-3^; not corrected for phylogenetic correlations). Similarly, body length of pollinating wasps was distributed into two size categories (Figure 5B): wasp genera that pollinated fig trees from a single subsection (i.e. *Agaon, Paragaon, Courtella *and *Allotriozon*) were significantly larger than genera pollinating several plant subsections (*t *= 7.43; *p *< 10^-3^; not corrected for phylogenetic correlations). Graphically, genus *Paragaon *presented small-sized wasps and therefore escapes this pattern. However, this genus includes only two species and for only one of them is the host known. Wasp size in this genus is thus little informative.

## Discussion

### The monophyly of section *Galoglychia*

Rønsted *et al. *[33] did not demonstrate the monophyly of the African section *Galoglychia*. Based on ITS+ETS data, they suggested that section *Galoglychia *might be paraphyletic to the American section *Americana*, although this result was ambiguously supported. With our ITS+ETS data set, all three types of analysis (MP, ML and BI) also failed to resolve the placement of section *Americana *relative to section *Galoglychia*, although a 3 bp deletion in the ETS sequence characterises section *Americana *and a 1 bp insertion in the ITS sequence characterises section *Galoglychia*. However, the chloroplast phylogeny provides strong support for the monophyly of section *Galoglychia *with each of the three methods. Moreover, a 10 bp insertion and a 1 bp deletion in fragment *FcL *characterise species of section *Americana *and a 10 bp insertion in the same gene characterises species of section *Galoglychia*. Hence the phylogeny based on chloroplast data enables to resolve the trifurcation of the phylogeny based on ITS+ETS data and the result is further supported by insertion-deletion data which were not taken into account in the phylogenetic reconstructions. Eventually, our chloroplast data set provided less information on terminal nodes, but offered better resolution for deeper nodes. Because chloroplast genes overall evolve more slowly than nuclear genes do [43], they are less prone to homoplasy and are therefore usually recommended for deep phylogenies [44-46].

Morphologically, sections *Galoglychia *and *Americana *share the presence of two bracts subtending the figs as opposed to generally three in genus *Ficus*. Section *Galoglychia *is characterised by all the bracts of the ostiole (the passage leading into the fig) turned inward, the orifice of the ostiole forming a bilabiate slit. In section *Americana*, the bracts of the ostiole are imbricate so that generally three bracts are visible as closing the ostiole. The monophyly of section *Galoglychia*, as recognized by botanists since the major revision of African figs ([47]; recognized as subgenus *Bibracteatae *at that time), is hence most probably correct.

### The infrasectional classification of section *Galoglychia*

The nuclear phylogeny proposes the division of section *Galoglychia *into two clades that do not include the same subsections as the two clades evidenced by the chloroplast analyses. Although the two nuclear clades are weakly supported (as previously observed by Rønsted *et al. *[33], several features suggest that this phylogeny is reliable. First, the alternative topology suggested by the chloroplast data set was demonstrated to be significantly less likely than the nuclear topology. Second, although 35% of our taxa were different from those used by Rønsted *et al.*, we found a similar topology with similar values of bootstrap supports. It is thus unlikely that the sampling composition (which covers half of the species of section *Galoglychia*) biased the phylogenetic reconstruction. Third, the position of most species remains consistent with classical taxonomy [18]. A pattern seems to appear in the taxonomic modifications revealed by molecular phylogenies in *Ficus*, suggesting that they do not result from identification/manipulation errors or from limiting phylogenetic information. Indeed, within subsection *Platyphyllae*, *F. platyphylla *and *F. jansii *both present clusters of small figs at the axils of the leaves or just below, a trait they share with a number of species of subsection *Crassicostae*, with which they are grouped in the phylogenetic tree. Conversely, *F. glumosa *and *F. stuhlmannii *only present two figs at the axils of the leaves and are grouped with species from subsection *Chlamydodorae*, which all have figs in pairs at the axils of the leaves. Preliminary data on some other species of subsection *Platyphyllae *seem to support this splitting of the species into two different units, mainly according to this trait. Fourth, a potential source of error using ITS and ETS could be the amplification of paralogous copies of ITS and ETS leading to an incorrect phylogenetic reconstruction [48-50]. However, here again, the monophyly of each subsection is hardly compatible with the amplification of paralogous copies of ITS and ETS that generally evolve independently [51]. Four events of extinction following the duplication would have to be hypothesised to obtain an observed nuclear topology corresponding to the chloroplast topology (Figure 4). Although this number of duplication-extinctions can reasonably occur given the considered time lapse [39], these four events would have had to occur in the ancestor of each of the four extant subsections and not within the subsections, a pattern which is unlikely. Last, the separation of section *Galoglychia *into two nuclear clades is supported by ecological and biogeographical arguments [33]. All these lines of evidence lead us to conclude that subsections *Galoglychia*, *Cyathistipulae*, *Crassicostae*, *Caulocarpae *and part of *Platyphyllae *on one hand, and subsection *Chlamydodorae *and part of *Platyphyllae *on the other hand, form two monophyletic clusters.

### A discordant chloroplast topology

Our investigation of the overall discordance between the chloroplast and the nuclear topology revealed two types of discordances occurring at two taxonomic levels. The first type concerns phylogenetic relationships between subsections: subsections *Cyathistipulae *and *Crassicostae *branch with subsections *Chlamydodorae *and *Platyphyllae *in the chloroplast topology, and with subsections *Galoglychia *and *Caulocarpae *in the nuclear topology. As highlighted above, the nuclear topology is consistent with morphological and biogeographical data. We can thus confidently assume that it is the chloroplast and not the nuclear topology that is discordant with the species tree. The second type of discordance concerns the monophyly of subsections: *Chlamydodorae *and *Platyphyllae *are paraphyletic in the chloroplast phylogeny (at least for *F. craterostoma *and *F. trichopoda*), and monophyletic in the nuclear phylogeny. The monophyly of subsections found in the nuclear tree is consistent with the morphological traits that led botanists to recognize these subsections for a century [47], meaning that here again, the nuclear but not the chloroplast phylogeny is more likely to reflect the species tree.

Examples of cyto-nuclear discordance are rather common in plants [52-57] and a limited number of causes leading to such discordances has been identified. Discordance between cytoplasmic and nuclear phylogenetic trees may either be artefactual or evidence distinct evolutionary histories followed by cytoplasmic and nuclear genomes [39]. Long Branch Attraction (LBA hereafter; [58]) is a source of artefact that may generate cyto-nuclear discordance [59,60]. Several arguments allow us to exclude LBA artefacts for our data set. First, the branches leading to discordance are not the longest branches of the tree, while some longer branches do not have unexpected positions in the tree. Further, the molecular data sets are not saturated which means that our markers did not evolve rapidly enough for phylogenetic signals to be lost. Finally, the discordance was the same whatever the phylogenetic reconstruction method employed. Although methodological concordance does not rule out LBA [61], parsimony has a stronger bias towards grouping long branches together than ML and BI which take into account unequal rates or branch lengths [62,63]. It is therefore unlikely that the observed cases of discordance resulted from LBA.

The cyto-nuclear discordance has therefore to be explained in terms of difference between species and chloroplast history. Two mechanisms may be involved: incomplete chloroplast lineage sorting and introgression. Differentiating between hypotheses of introgression and lineage sorting is difficult because both processes can generate very similar phylogenetic patterns [64]. Ancestral polymorphism is expected to be completely sorted out with a high probability approximately 4*N*_*e *_generations (*N*_*e *_= effective population size) after the separation [65]. The branch supporting the CCCP clade (BL = 0.1774) is longer than the branch supporting clades in which reciprocal monophyly has been reached, like the *Caulocarpae *clade (BL = 0.1699), the *Galoglychia *clade (BL = 0.1269) and section *Americana *(BL = 0.1756). Hence, this branch is expected to be long enough to have allowed chloroplast genes to reach reciprocal monophyly as well and hence to complete allele sorting. However, the sorting depends on *N*_*e *_and not on branch length directly. Therefore, any variation in *N*_*e *_over time and across species would alter conclusions based on relative branch length comparison. Methods based on coalescent simulations to calculate the probability of observing lineage sorting are also based on *N*_*e *_remaining constant over time and among species [66] and both hypotheses are not realistic when comparing savannah and forest lineages over longer periods of time. Moreover, they require estimating ancestral population sizes that can only be obtained by intensive intraspecific sampling and this is not compatible with the phylogenetic approach used here. Eventually, these methods require generation time to be known and do not allow this parameter to vary across the phylogeny. *Ficus *lineages included in our phylogeny are highly different in term of height and life forms, with some lineages represented mainly by canopy emergent trees (e.g. subsection *Caulocarpae*) and other mainly by small shrubs and trees in open habitats (e.g., subsection *Chlamydodorae*). Because the generation time is known to vary with life form and height in trees [67], substantial variation in the generation time across the *Galoglychia *phylogeny would generate strong uncertainty for results based on coalescent methods. In conclusion, because of too many uncertainties in the inputs required for more subtle models, we assess that the method based on relative branch lengths, although not optimal, is appropriate to discuss lineage sorting with the current data set. Although we are not able to fully exclude it, the incomplete lineage sorting hypothesis thus does not appear a likely explanation for the observed cyto-nuclear discordance.

### Host shift mediated introgression as the cause of phylogenetic discordance

Introgressive hybridization, i.e. the introduction of an allele inside the genome of a foreign taxon after hybridization [13], could explain the observed pattern. Introgressive hybridization in *Ficus *necessitates that a pollinating wasp fertilizes two hosts. Hybridization may result from sporadic visitation of an alternative host by a wasp, and may in that case not require successful development of wasp offspring. However, it may also result from transient or even stabilised utilisation of two hosts by a pollinating wasp species, and could result in subsequent speciation of the wasps on the new host. Thus, pollinator taxonomy and phylogeny may be informative to analyse whether introgressive hybridization could account for discrepancies between markers in the *Ficus *phylogeny.

**Figure 6:**
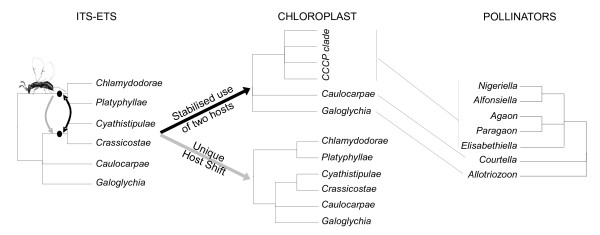
**Comparative phylogeny and the host shift scenario**. If we accept current phylogenies, then a pollinating wasp of an ancestor of *Chlamydodorae *and *Platyphyllae *must have shifted to an ancestor of *Cyathistipulae *and *Crassicostae*, an event required to explain the discrepancy between ITS+ETS (left) and pollinator (right) phylogenies. However, under the hypothesis of a wasp suddenly changing host (grey arrows), there is no cytoplasm transfer and the *Ficus *lineage resulting from the hybridization necessary bears a chloroplast related to the *Caulocarpae *and *Galoglychia *lineages (middle bottom), a feature which is not observed in the cytoplasm phylogeny. On the contrary, if a wasp uses two hosts for a prolonged period of time (black arrows), repeated backcrosses may allow the introduction of a chloroplast originating in the *Chlamydodorae *plus *Platyphyllae *lineage inside the ancestor of *Cyathistipulae *and *Crassicostae *(middle top), as observed in the data set. The set of phylogenetic trees therefore support the role of an intermediate stage in which a pollinating wasp has consistently used two host species.

Subsections *Cyathistipulae *and *Crassicostae *present discordant positions between the chloroplast and nuclear phylogenies. Subsection *Cyathistipulae *is strictly associated with genus *Agaon*. The phylogenetic analysis concludes to nuclear monophyly and does not reject cytoplasmic monophyly. On the other hand subsection *Crassicostae *is pollinated by what could be its own genus of pollinators, *Paragaon *(two species known, and host association only known for one species) but also more frequently by *Elisabethiella *and in one case by *Nigeriella*, i.e. genera also associated with subsections *Chlamidodorae *and *Platyphyllae*. Hence this part of the cyto-nuclear mismatch could be associated with repeated pollinator host shifts. Erasmus *et al. *[26] published a phylogeny of wasps pollinating *Ficus *species of section *Galoglychia *that may shed light on the origin of genera *Agaon *(the exclusive pollinators of subsection *Cyathistipulae*) and *Paragaon *(pollinating some species of *Crassicostae*). Based on a combined ITS/28S data set, they found the following topology: (((*Agaon *&*Paragaon*), (*Alfonsiella *&*Nigeriella*), *Elisabethiella*), *Courtella*, *Allotriozoon*). Hence, the cytoplasmic CCCP clade is exclusively pollinated by the *Agaon*, *Paragaon*, *Alfonsiella*, *Nigeriella *and *Elisabethiella *clade. One may therefore suggest that a first host shift of a pollinator from an ancestor of the *Chlamydodorae *to an ancestor of the *Cyathistipulae *and *Crassicostae *may have given rise to the *Agaon*-*Paragaon *lineage (detailed in Figure 6). This would be the first and major event bringing about the cyto-nuclear discordance. Then we need to hypothesize a second wasp transfer between these two large clades, from *Chlamydodorae *to *Crassicostae*. In this scenario, the pollinators bring with them the cytoplasm of their original host. A simple way to achieve such an a priori unexpected cytoplasm transfer is the stabilised occurrence of a species of fig pollinating wasps using two host species. Such a situation allows backcrosses of hybrids with both parental species. Indeed field observations report several cases of two *Ficus *species being pollinated by the same wasp species [26]. For instance *Elisabethiella stuckenbergii *is known to pollinate *F. burkei*, *F. natalensis natalensis*, *F. lingua depauperata *and *F. petersii *[32], a feature which could potentially allow cytoplasm transfer in any direction independently of which of these *Ficus *species, if any, was the original host of the wasp.

The scenario presented above hinges on the relative phylogenetic position of the different genera pollinating section *Galoglychia*. A recently published pollinator phylogeny [68] proposed that pollinators of subsections *Chlamydodorae, Platyphyllae, Cyathistipulae *and *Crassicostae *are monophyletic, as in the phylogeny of Erasmus *et al. *[26], and thus does not contradict the scenario proposed above. This new phylogeny did not include *Allotriozoon *and *Paragaon *and did not fully resolve the position of the different genera.

The second type of discordance, concerning the monophyly of subsections, suggests that introgressive hybridization has occurred inside the CCCP clade, with at least two evidenced instances. Both cases concerned species from subsections that are pollinated by several wasp genera. All wasp phylogenies demonstrated the monophyly of wasp genera [26,68]. Taken together, these observations support the hypothesis that hosts shift could account for several wasp genera pollinating a single *Ficus *subsection. Erasmus *et al. *[26] and Jousselin *et al. *[36] recently suggested that original pollinator species duplication followed by more recent extinction of one or other of the lineages in host figs, rather than host shifts, could explain this unusual association pattern. Beyond evidence for cytoplasmic gene transfers in our phylogenies, there are two reasons that do not favour this hypothesis. First we would need to apply this line of reasoning to subsection *Crassicostae *which is pollinated by three wasp genera (*Paragaon*, *Elisabethiella*, *Nigeriella*). As *Elisabethiella *and *Nigeriella *also pollinate subsection *Chlamydodorae*, these genera should then have differentiated into well defined genera very early in section *Galoglychia *history and only much later would one genus have become extinct in some species or even subspecies and survived in other species or even subspecies. The second reason is that Erasmus *et al. *[26] provided direct evidence of ongoing local pollinator exchange between *Ficus *species. Indeed *Alfonsiella binghami *samples from Nelspruit (South Africa), formed a single genetic group whether collected on *F. petersii *or on *F. sthulmannii *comparatively to samples of *A. binghami *also collected on *F. sthulmannii *but in Lekgalameetse (South Africa) and in Amani (Tanzania). Finally, recovering a phylogenetic signal suggesting some amount of parallel cladogenesis is not sufficient to infer that parallel cladogenesis is relevant within these taxonomic groups. Indeed, if host shifts preferentially occur between closely related hosts [69], then the limited amount of evidence suggesting host-pollinator co-cladogenesis in section *Chlamydodorae *[36] could easily result from host shifts. Hence the evidence in favour of the occurrence of host shifts within the pollinators of part of section *Galoglychia *is overwhelming.

### Fig size: a factor facilitating host shifts?

Although we statistically demonstrated only three instances of cyto-nuclear discordance, it is remarkable that these signs of introgressive hybridization were only found in subsections that are implied in the incongruence between plant and insect phylogeny and taxonomy. If these results strongly supports the hypothesis that host shift is the cause of this incongruence, it also stresses the fact that host shifts have occurred preferentially in some particular lineages of section *Galoglychia*. Hence, host shift could have been facilitated by particular phenotypic traits in wasps or host plants rather than resulting from purely stochastic processes. When we compare the six subsections of section *Galoglychia*, a striking pattern emerges. All subsections that share pollinator genera and present cytoplasmic introgression also present, on average, small figs and are pollinated by small wasps. Conversely all subsections that have their own chloroplast lineage (or for which cytoplasm monophyly cannot be ruled out) and are pollinated by their own genus of agaonid wasps produce large figs and are pollinated by large wasps. Among pollinators of section *Galoglychia*, large wasp species seem therefore to be more host-specific than small wasp species, a pattern of association that could correlate with a simpler and shorter ostiole passages into small figs of section *Galoglychia*. Indeed, in Gabon, Michaloud [70] showed that *Courtella sp. *pollinating *Ficus ottoniifolia *(subsection *Caulocarpae*) looses its wings and antennae on entering the tight ostiole of the fig, while *Alfonsiella fimbriata *pollinating *F. natalensis leprieuri *(section *Chlamydodorae*) only looses its wings and retains its antennae, going through an ostiole that Michaloud describes as a simple slit.

## Conclusion

As predicted by the hypothesis that host shifts are responsible for the lack of congruence between insect and plant phylogeny and taxonomy in section *Galoglychia*, we found evidence for several cases of chloroplast genetic introgression events. The alternative to host shifts would be duplication of insect taxa on a host followed by later extinction. Separating these two hypotheses is extremely difficult [69,71]. However, in our case, because two genetically independent markers, nuclear DNA carried by insects and chloroplasts, tell the same story, the hypothesis of host shifts becomes much more likely. Knowing which lineages are involved in host shifts within a *Ficus *section allowed us to infer that a facilitating factor for host shift could be small fig size. Detecting identical or analogous patterns to those described here in other sections of the genus will constitute the real test of their generality. Finally, while chloroplast DNA presents limited variability in *Ficus *[72], our initial screening of markers allowed increasing the number of polymorphic sites. We were eventually able to resolve deep nodes in the *Ficus *phylogeny for which ITS and ETS nuclear markers were not sufficiently informative. Non-coding chloroplast markers could thus be very helpful to resolve basal branching inside the *Ficus *phylogeny; a necessary step for an in depth analysis of the history of the coevolution between figs and their pollinating wasps.

## Methods

### Taxon sampling

Forty four specimens belonging to 38 species of *Ficus *out of 77 from the section *Galoglychia *were analysed in addition to 10 species from section *Americana*. *Ficus carica, F. ingens, F. variegata *and *F. orthoneura *were chosen as outgroups because they belong to distinct sections originating from Asia. This sampling encompasses all six subsections of section *Galoglychia *sensu Berg [31] and confirmed by Rønsted *et al. *[33]. For six species, two specimens from different locations were collected and used for validation. Most of the material consisted of dried leaves collected during field work mainly by FK, from herbaria and from living collections. Forty-one ITS and 33 ETS accessions were retrieved from GenBank (following previously published papers [5,22,33]). We chose to acquire our own set of nuclear sequences for species for which available sequences came from individuals of unknown geographic origin and belonging to species with large pan-african distribution ranges, or for species recognized to include several subspecies or morphotypes. This procedure allowed us to be confident that we were comparing chloroplast and nuclear genes of the same taxa. A list of taxa with origin and GenBank accession numbers is provided in Additional file 2.

### Chloroplast DNA laboratory work

Although chloroplast markers were used in early *Ficus *phylogenetic studies [20], their low level of polymorphism and the associate difficulty to obtain resolved phylogenies down to genus level excluded them from subsequent studies [72]. We therefore developed our own chloroplast primer pairs. We focused on non-coding to select informative sequences. The detailed protocols used during molecular laboratory works are provided in Additional file 5. We designed primer pairs in *Ficus *using the Oligo4 software. The Staden Package was used to compile contiguous sequences of each accession and all polymorphic sites were checked against the original electrophoregrams. They were re-examined and adjusted manually using BioEdit [73]. To identify the position of *Ficus *sequences, they were blasted with the completely sequenced chloroplast genome, phylogenetically closest to *Ficus*, namely that of *Morus indica *[74]. Pairwise *P*-distance and average value with standard error estimated by 500 bootstraps were computed using the MEGA software version 3.1 [75].

Using universal primers developed by Grivet *et al. *[76] in the large single copy (LSC) region of chloroplast genome, we tested 25 amplifying DNA fragments smaller than 3 Kb on a sub-sample of 9 species (see Additional file 6) representing genus *Ficus *diversity with 6 sections and 3 species within section *Galoglychia*. We also tested two previously described sequences, *atpB-rbcL *[77] and *trnL *(UAA)3'exon-trnF(GAA) [78]. Based on the resulting sequences and their polymorphism, we selected 10 primer pairs and defined new primer pairs to amplify potentially informative sequences shorter than 1 kb (see Additional file 1). For the 3 fragments longer than 1 kb, we designed 2 to 3 primer pairs focusing on the most informative sequences with 6 to 15 substitutions. These 14 amplified sequences were distributed throughout the LSC chloroplast genome. We examined the amount of phylogenetic information conveyed by each marker on 33 *Ficus *species including 19 from sections *Galoglychia *and *Americana*. Finally, we eventually selected five markers on the basis of the phylogenetic information they conveyed: *atpB-rbcL, FcB, FcL, FcJ *and *trnL-trnF*.

### ITS and ETS laboratory work

Based on 100 ITS sequences previously published [5,22,33], we designed new primer pairs specifically defined in *Ficus *using the Oligo4 software. The primers ITSF1 (ACAAGGTTTCCGTAGGTGA) and ITSF4 (GTATAGTTATTCGCCTCCT) were defined as forward and reverse primers in 18S and 26S ribosomal RNA genes, respectively. An additional reverse primer ITSF5 (CGGAGGTTACGCTGGGGTC) was defined in the zone at the junction between ITS2 and 26S conferring a high specificity in *Ficus *species. Indeed, ITS amplification using the ITSF1-ITSF5 primer pair was selective and successful for some samples producing a single, clearly defined, DNA fragment. For the remaining samples, we used the ITSF1-ITSF4 primer pair that amplified several bands since they were defined on conserved sequences. For samples which produced several amplified DNA fragments, we selected the one presenting the expected size based on previous published ITS sequences [22,72] by isolating it from the agarose gel following a long electrophoresis migration. We then performed a second PCR on this isolated DNA fragment for sequencing. ETS amplification was performed using the ETS-Hel-1/18S-ETS primer pair [79].

### Alignment and phylogenetic analyses

Sequence alignment was performed using the program Clustal X [80] with manual adjustment and excluding ambiguous alignment positions (0.2% and 3.5% of the original chloroplast and nuclear data sets, respectively). All analyses with the chloroplast genes were performed with the five markers combined into a single matrix. This concatenation is not expected to modify the inference of the chloroplast history since this organelle is not known to recombine in wild Angiosperms [81]. For nuclear analyses, we followed previous studies that demonstrated that ITS and ETS sequences could be combined into a single matrix in *Ficus *[5,22]. In maximum parsimony (MP) analyses, all characters were equally weighted and treated as unordered. Heuristic searches using PAUP* 4b10 [82] were started with 1000 stepwise random addition sequence replicates, holding 10 trees at each step, followed by tree bisection-reconnection branch (TBR) swapping, saving maximally 100 most parsimonious trees. All shortest trees retained in memory were used as starting trees for a second round of searching using TBR branch swapping until all trees were found or a pre-set maximum of 15,000 trees were found. We eventually computed the strict consensus of the most parsimonious trees (SCMP). Relative levels of homoplasy in both nuclear and chloroplast data sets were assessed from all characters using the consistency index (CI) and the retention index (RI) as implemented in PAUP. Bootstrap support was assessed using 1000 replicates each consisting of 10 random addition sequence replicates with TBR swapping and no limits on the number of trees saved.

Bayesian inferences (BI) were performed with several partitioning strategies for each data matrix. Three strategies were tested with the chloroplast matrix: all chloroplast data combined (*P*_1 _in Table 1), one partition each for the five markers (*P*_2_-*P*_6_) and one partition each for the coding and non-coding DNA (*P*_7_-*P*_8_). Two strategies were tested with the nuclear matrix: all chloroplast data combined (*P*_9_) and one partition each for ITS and ETS (*P*_10_-*P*_11_). The appropriate model of sequence evolution for each partition was determined using the likelihood ratio test (LRT) implemented with MrModeltest [83].

We tested every partitioning strategy with BI with the program MrBayes 3.1.2 [84]. Model parameters were always taken as unlinked and the rate multipliers set as variable across partitions. Initial runs were conducted starting with random neighbour joining trees to check the number of simultaneous Markov Chain Monte Carlo (MCMC) chains necessary not to get caught on local optima. A Dirichlet distribution was assumed for the rate matrix and base frequency and every tree topology was assumed to be equally probable. The MCMC process was set so that two simultaneous independent analyses starting from different random trees with four chains (three heated) ran simultaneously over 4,000,000 generations, and every 200th tree saved into a file. Variation in likelihood scores was examined graphically to determine apparent stationarity for each independent run resulting in a burnin of 10% trees. MCMC convergence was also explored by examining the Potential Scale Reduction Factor (PSRF) convergence diagnostics for all parameters in the model. Finally, we determined posterior probabilities of the phylogenies and associated branches based on the stationary trees pooled from the two runs for each analysis.

The results for each partitioning strategy were compared to the strategy with the best arithmetic mean of the likelihoods (-lnL; sampled from the posterior) using Bayes factors (see [85] for details). Bayes factors were approximated by the ratio of the harmonic means of the likelihoods of the two strategies being tested [86]. Arithmetic and harmonic means were calculated using the *sump *command of MrBayes. In this study, we used the traditional cutoff criterion of 2ln Bayes factor of ≥ 10 as very strong evidence against the compared strategy [87].

Maximum likelihood (ML) analyses were performed with RaxML [88] which offers the possibility to partition data. For each data set, we therefore ran analyses using the best partitioning strategy and model of sequence evolution calculated with MrModeltest. We replicated 200 heuristic searches using a randomized maximum parsimony tree as starting tree. The confidence of branching was assessed using 1000 non-parametric bootstrap resamplings generated as heuristic searches. The information from the 1000 bootstrapped trees was drawn on the best-scoring ML tree from the 200 runs.

Gap characters representing mainly deletions were studied separately from the nucleotide matrices. Gaps were coded for each data set using SeqState [89] with the Simple Indel Coding approach (SIC) [90]. This method scores all gaps, regardless of length, as separate presence/absence characters. Sequences with gaps that extend beyond both the 5'- and 3'-termini of the gap being coded, as well as sequences with gaps that extend beyond one terminus and to the other terminus, are scored as missing data for that character. We used preferentially the SIC approach because it has been demonstrated to outperform the classical method consisting in coding gaps as 5th states for each and every nucleotide position regardless of length [91]. We eventually mapped gaps onto the topologies resulting from the analyses of the respective data sets. Because of limited space along branches, we mapped only synapomorphic gaps.

### Analyses of cyto-nuclear discordances

Chloroplast and nuclear phylogenies appeared discordant for several internal and external nodes. The nested pattern of the discordances prevented us from testing their individual significance by using the classical method of removing all discordant taxa and then adding them individually in comparative tests (see [39] for an example). Instead, we used Templeton tests [92] with trees modified from the SCMPs, and Shimodaira-Hasegawa tests (SH; [93]) with trees modified from the ML trees. SH tests were implemented in PAUP, using the REEL approximation with 10,000 bootstrap replicates. Because PAUP cannot account for partitions, models of sequence evolution specified in SH tests were those calculated with all data combined for each data set. Basically, for two phylogenetic hypotheses to be discordant, a topology A must explain significantly better a data set A than a topology B does it, and a topology B must explain significantly better a data set B than a topology A does it. Both conditions need to be full-filled to be able to assess the significance of a given discordance. For example, the global discordance between nuclear and chloroplast topology was tested by comparing the length of the SCMP of the chloroplast trees with the length of the nuclear SCMP, based on the chloroplast data set. The test was then repeated based on the nuclear data set. Similarly, we tested several local discordances individually by comparing a SCMP topology with the same SCMP topology in which only one discordant accession was moved manually in the position as found in the rival topology; each time duplicating the test with both data sets. Last, we tested discordances with a Bayesian approach [85]. This was achieved by first building 95% credible sets of unique trees (sampled at stationarity) with the *sumt *command in MrBayes for each the chloroplast and nuclear analysis. Then, we used the SumTrees [94] software to check for the presence of alternative phylogenetic hypotheses in the 95% credible set. If they were absent, these hypotheses could be rejected statistically.

Because saturation in substitutions can lead to incorrect phylogenetic inferences [95], we plotted the estimated number of transitions and transversions against the genetic distance using DAMBE V 4.5.32 [96]. In an unsaturated data set, transitions and transversions are both expected to increase linearly with the genetic distance, with a steeper slope for transitions than for transversions. Analyses were conducted with chloroplast, ITS and ETS sequence matrixes separately.

We investigated the hypothesis of amplifying paralogous copies of nuclear genes by assessing the minimum number of gene duplications and extinctions that are required to generate the observed discordant pattern with the help of the GeneTree 1.3.0 software [97,98]. This procedure was only done for the nuclear genes because gene duplication has not been documented for plastid loci [99]. Because ITS and ETS belong to the same unit of transcription, we did not separate them for this analysis.

### Fig and pollinator size

Data on fig and pollinator size were retrieved from Berg & Wiebes [18]. As the data were collected by the same observers, the values were comparable. Dry fig diameter was averaged for each subsection of section *Galoglychia*. We used diameter of dry figs because it was the sole fig-size measure available for all species. We also used body length for each pollinator wasp species. Body length included head, thorax and gaster but excluded the ovipositor. Simple mean comparison tests were performed to compare fig/wasp size between subsections/genera involved in strict association with their partners and subsections/genera involved in multiple associations.

## Authors' contributions

JPR, FK and BK jointly designed and coordinated the study. FK carried out the sampling and BK, CG and SS the laboratory analyses. JPR analysed the sequence data sets and wrote the first draft of the manuscript. FK and BK finalized the manuscript. All authors read and approved the final manuscript.

## Supplementary Material

Additional file 1 **Description of the new chloroplast primer pairs specifically defined in *Ficus***. This table provides basic information about primers like name, sequence and position in *Morus indica *genome. Click here for file

Additional file 2 **List of samples included in the final analyses with origin and GenBank accession numbers**. This table provides species name, origin, voucher number and GenBank accession number for samples. Click here for file

Additional file 3 **Insertions and deletions in chloroplast and nuclear markers**. This data provides information about position and length for indels. Click here for file

Additional file 4 **Substitution pattern of the chloroplast markers, ITS and ETS genes**. The number of transitions and transversions is plotted against the TN93 distance. Click here for file

Additional file 5 **Protocols for molecular laboratory works**. A text providing details for extraction, amplification and sequencing procedures. Click here for file

Additional file 6 **List of species used for the development of non coding chloroplast DNA markers for *Ficus***. A table giving voucher numbers and origins. Click here for file
