# Supplementary material for: Cyto-nuclear discordance in the phylogeny of Ficus section Galoglychia and host shifts in plant-pollinator associations
Source: BMC Evol Biol. 2009 Oct 12;9:248. doi: 10.1186/1471-2148-9-248 (PMC2771017; doi:10.1186/1471-2148-9-248)
Supplement: Additional file 1 — Description of the new chloroplast primer pairs specifically defined in Ficus. This table provides basic information about primers like name, sequence and position in Morus indica genome. [file 1471-2148-9-248-S1.DOC]

**Additional file 1.** Description of the new chloroplast primer pairs specifically defined in *Ficus* on the basis of producing potentially phylogenetically informative characters.

| cp consensus primers pairs | Sequence name | Primer | Sequence (5’-3’) | Size (bp) | Position in *Morus indica* | PICs^4^ | | Single subst. | P-distance | SE |
| --- | --- | --- | --- | --- | --- | --- | --- | --- | --- | --- |
|  |  |  |  |  |  | Subst.^5^ | Indels^6^ |  |  |  |
| 7^a^-*trnR*^b^-*ccmp4-U*^c^ | *FcI* | FC-21F^1^ | TCACCTAAACGAGAACTTG | 627 | 11527 - 12153 | 4t^7^, 1tv^8^ | 0 | 5 | 0.00351 | 0.00137 |
|  |  | FC-22R^2^ | GGCTTTTTTGGGTCGTGTT |  |  |  |  |  |  |  |
|  | FcJ | *FC-23F* | AATGCCATCGCCTACCTGA | 756 | 12342 - 13094 | 5t, 3tv | 4 | 5 | 0.00243 | 0.00082 |
|  |  | *FC-24R* | TATGAATAGAGGACAGGCT |  |  |  |  |  |  |  |
| 9 ^a^ -*atpH*^b^-*atpI*^c^ | *FcA* | *FC-05F* | CGACGCCCCCCGATGAATT | 632 | 14867 - 15497 | 3t, 5tv | 5 | 18 | 0.00456 | 0.00131 |
|  |  | *FC-06R* | TTCACAATAGTAGGGCAAA |  |  |  |  |  |  |  |
| 12 ^a^ -*rpoC2*^b^-*rpoC1*^c^ | *FcK*^f^ | *FC-25F* | CCTTGTGGGTCCGACATTAATCCTC | 1488 | 21327 - 22807 | 5t^d^ | 0^d^ | 7^d^ | 0.00235 | 0.00075 |
|  |  | *FC-26R* | GCGAAATAGCAATAGAACT |  |  |  |  |  |  |  |
| 13 ^a^ -rpoC1^b^-rpoB^c^ | FcL | *FC-27F* | CTGGTAATAGCACAAAACCCATCC | 781 | 23204 - 23974 | 8t, 3tv | 4 | 10 | 0.0044 | 0.00127 |
|  |  | *FC-28R* | AAGATTGAATGGAACGATA |  |  |  |  |  |  |  |
| *TrnL3’-trnF*^d^ | *FcM* | *FC-29F*^3^ | TCCGTCGACTTTAGAAATCGTG | 478 | 50592 - 50898 | 6t, 2tv | 0 | 10 | 0.00536 | 0.00177 |
|  |  | *FC-30R*^3^ | TGCCAGGAACCAGATTTGAACT |  |  |  |  |  |  |  |
| *atpB-rbcL*^e^ | *atpB-rbcL*^e^ | *S2r-F*^e,3^ | AGAAGTAGTAGGATTGATTCTCATA | 773 | 56761 – 57148  57166 - 57462 | 5t, 3tv | 6 | 12 | 0.0029 | 0.00076 |
|  |  | *rbcL1-R*^e,3^ | GAATCCAACACTTGCTTTAGTCTCT |  |  |  |  |  |  |  |
| 31 ^a^ -*rps18*^b^-*clpp*^c^ | *FcF* | *FC-15F* | TATTGTTGTGTCGTAAGAA | 610 | 71402 - 71993 | 3t, 1tv | 0 | 7 | 0.00283 | 0.0011 |
|  |  | *FC-16R* | AACTGAAAGAAAAGAAATC |  |  |  |  |  |  |  |
|  | *FcG* | *FC-17F* | CTTTCACAACGAGACCACT | 514 | 72364 - 72835 | 3t, 1tv | 2 | 9 | 0.00305 | 0.00121 |
|  |  | *FC-18R* | AACACAAGACAGCCAATCA |  |  |  |  |  |  |  |
|  | *FcH* | *FC-19F* | GCTGTCTTGTGTTTCTAAT | 405 | 72824 - 73228 | 10t, 4tv | 1 | 8 | 0.01777 | 0.00433 |
|  |  | *FC-20R* | AACCTGCTAGTTCTTTTTAT |  |  |  |  |  |  |  |
| 33 ^a^ -*psbB*^b^-*psbB*^c^ | *FcC* | *FC-09F* | TGTTCGTTATACCCTTCAT | 877 | 75816 - 76655 | 5t | 0 | 9 | 0.00275 | 0.00106 |
|  |  | *FC-10R* | CACGCCCTTCTTTATCTCT |  |  |  |  |  |  |  |
| 34 ^a^ -*psbB*^b^-*petB*^c^ | *FcD* | *FC-11F* | ACATTCCTCTTAGTCTCAA | 529 | 77364 - 77891 | 2t, 2tv | 1 | 7 | 0.00384 | 0.00156 |
|  |  | *FC-12R* | CAATACCCATAAAAGGAGT |  |  |  |  |  |  |  |
|  | *FcE* | *FC-13F* | ATTGACTCATCGTGGTTCT | 731 | 78503 - 79235 | 2t, 4tv | 0 | 10 | 0.00345 | 0.0012 |
|  |  | *FC-14R* | TAAAGCCACCAGTGAGATA |  |  |  |  |  |  |  |
| 35 ^a^ -petB^b^-petD ^c^ | FcB | *FC-07F* | ATTACCTCTTCTTACTGCC | 745 | 79474 - 80220 | 5t, 4tv | 3 | 9 | 0.00357 | 0.00098 |
|  |  | *FC-08R* | TCAAGTGCTTTCTGGGTCG |  |  |  |  |  |  |  |

^a^ No. of cp consensus primers pairs as indicated in Table 1 [76]

^b^ and ^c^ primer forward and reverse, respectively [76]

^d^developed by Taberlet *et al.* [78]

^e^described by Kress *et al.* [77]

^f^Screening based on 9 *Ficus* species

^1^Forward, ^2^Reverse

^3^Annealing temperature at 50° C, for all others primers pairs, the annealing temperature is 55 °C

^4^Potentially informative characters

^5^Substitutions, ^6^Insertion/deletion

^7^Transitions, ^8^Transversions
