# Supplementary material for: Cyto-nuclear discordance in the phylogeny of Ficus section Galoglychia and host shifts in plant-pollinator associations
Source: BMC Evol Biol. 2009 Oct 12;9:248. doi: 10.1186/1471-2148-9-248 (PMC2771017; doi:10.1186/1471-2148-9-248)
Supplement: Additional file 3 — Insertions and deletions in chloroplast and nuclear markers. This data provides information about position and length for indels. [file 1471-2148-9-248-S3.DOC]

**Additional file 3.** Insertions and deletions in chloroplast and nuclear markers. “#” is the reference number mapped onto Figure 2.

| *Chloroplast* | | | | |  | *Nucleus* | | | | |
| --- | --- | --- | --- | --- | --- | --- | --- | --- | --- | --- |
| # | Marker | Position | Length | Description |  | # | Marker | Position | Length | Description |
| 1 | *atpB-rbcL* | 540 | 14 | insertion |  | 1 | *ETS* | 987 | 1 | deletion |
| 2 | *FcL* | 2510 | 1 | deletion |  | 2 | *ETS* | 1012 | 3 | insertion |
| 3 | *FcL* | 2684 | 10 | insertion |  | 3 | *ETS* | 949 | 3 | deletion |
| 4 | *FcL* | 2672 | 10 | insertion |  | 4 | *ITS* | 345 | 1 | insertion |
| 5 | *FcB* | 1108 | 13 | deletion |  | 5 | *ITS* | 333 | 1 | insertion |
| 6 | *FcL* | 2687 | 7 | deletion |  | 6 | *ITS* | 284 | 1 | deletion |
| 7 | *atpB-rbcL* | 663 | 8 | deletion |  | 7 | *ITS* | 653 | 1 | deletion |
| 8 | *FcJ* | 2436 | 12 | insertion |  | 8 | *ETS* | 1228 | 2 | deletion |
| 9 | *FcL* | 2530 | 1 | deletion |  | 9 | *ETS* | 984 | 1 | deletion |
| 10 | *FcL* | 2822 | 12 | insertion |  | 10 | *ETS* | 1162 | 1 | insertion |
| 11 | *FcL* | 2501 | 1 | deletion |  |  |  |  |  |  |
| 12 | *atpB-rbcL* | 177 | 1 | deletion |  |  |  |  |  |  |
| 13 | *trnL-trnF* | 3472 | 1 | deletion |  |  |  |  |  |  |
