# Supplementary material for: Cyto-nuclear discordance in the phylogeny of Ficus section Galoglychia and host shifts in plant-pollinator associations
Source: BMC Evol Biol. 2009 Oct 12;9:248. doi: 10.1186/1471-2148-9-248 (PMC2771017; doi:10.1186/1471-2148-9-248)

**Additional file 4.** Substitution pattern of (A) the chloroplast markers, (B) the ITS gene and (C) the ETS gene. The number of transitions (crosses) and transversions (triangles) is plotted against the TN93 distance considering all sites. Each point represents a pairwise comparison between two taxa.

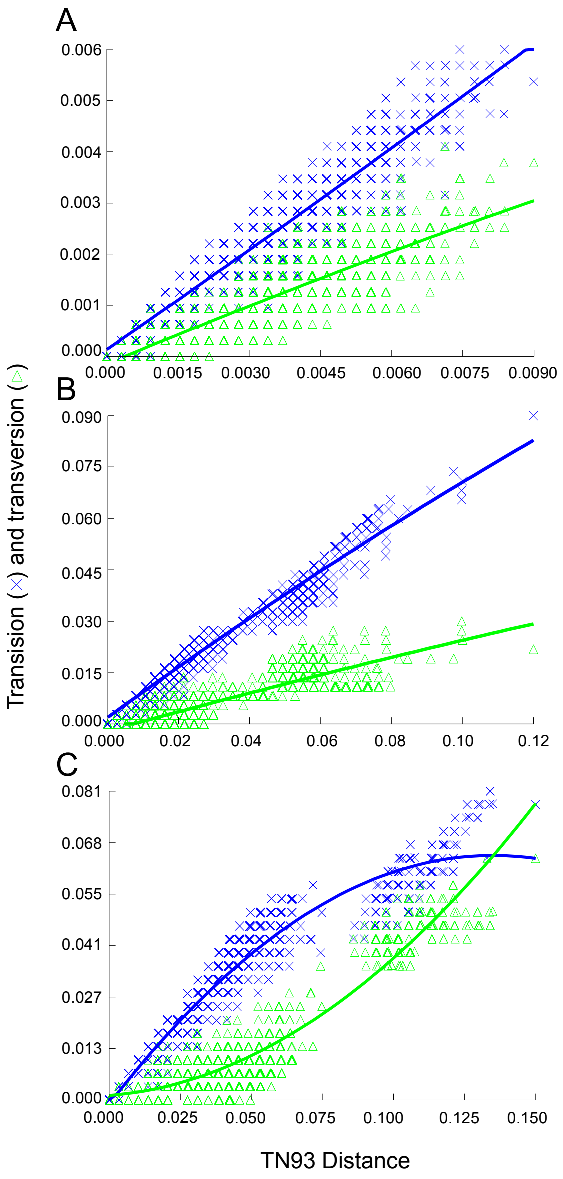

Supplement: Additional file 4 — Substitution pattern of the chloroplast markers, ITS and ETS genes. The number of transitions and transversions is plotted against the TN93 distance. [file 1471-2148-9-248-S4.PDF]
