# Supplementary material for: Cyto-nuclear discordance in the phylogeny of Ficus section Galoglychia and host shifts in plant-pollinator associations
Source: BMC Evol Biol. 2009 Oct 12;9:248. doi: 10.1186/1471-2148-9-248 (PMC2771017; doi:10.1186/1471-2148-9-248)
Supplement: Additional file 5 — Protocols for molecular laboratory works. A text providing details for extraction, amplification and sequencing procedures. [file 1471-2148-9-248-S5.DOC]

**Additional file 5. Protocols for molecular laboratory works. A. Chloroplast DNA laboratory work. B. ITS and ETS laboratory work.**

**A**. DNA was extracted from frozen fresh leaves (100 to 200 mg) or from silica dried leaves (50-100 mg) using the DNeasy Plant Mini Kit (Qiagen) with the following modification: 1% of Polyvinylpyrrolidone (PVP 40 000) was added to buffer AP1. Among the nine individuals previously studied in Rønsted *et al.* {Rønsted, 2007 #45}, we extracted DNA of two according to this protocol. For the remaining seven samples, we used total genomic DNA of already extracted these authors. Amplification reactions were performed in a final volume of 25μL in the presence of 1 to 5 ng of template DNA, 10 pmoles of each primer, 1x reaction buffer (10 mM Tris-HCl pH 9.0, 50 mM KCl, 0.1% TritonX100, 0.02% gelatin), 2 mM MgCl2, 0.2 mM of each dNTP and 0.5U Taq DNA polymerase (Sigma). The PCR method was as follows: 94°C for 6 mn, 35 cycles of 94°C for 30s, 50 or 55°C (depending of the primer pair) for 45s and 72°C for 45s; followed by a final extension for 10 mn at 72°C. PCR products were purified prior to sequencing with the AMPure PCR purification Kit (Agencourt). DNA sequencing was performed with the ABI PRISM BigDye Terminator Mix V3.1, using the thermal cycle parameters 92° C, 5mn; cycles of 30 s denaturation (92 °C), 20s anneling (50 °C), and 4 mn 30s elongation (60 °C). Products were run on an ABI PRISM 3130 XL semi-automated sequencer.

**B**. Amplification reactions were performed in a final volume of 25µL in the presence of 1 to 5 ng of template DNA, 5 (for ITS) and 10 (for ETS) pmoles of each primer, 1x reaction buffer (10 mM Tris-HCl pH 9.0, 50 mM KCl, 0.1% TritonX100, 0.02% gelatin), 2 mM MgCl2, 0.2 mM of each dNTP and 0.5U Taq DNA polymerase (Sigma). The PCR method was as follows: 94°C for 6 minutes, 35 cycles of 94°C for 30s, 50°C for 30s (ITS) to 60s (ETS) and 72°C for 2 min 30s; followed by a final extension for 10 minutes at 72°C. PCR products were purified prior to sequencing with the AMPure PCR purification Kit (Agencourt). DNA sequencing was performed with the ABI PRISM BigDye Terminator Mix V3.1, using the thermal cycles parameters 92° C, 5mn; 60 cycles of 30s denaturation (92 °C), 20s anneling (50 °C), and 4 mn 30s elongation (60 °C). Products were run on an ABI PRISM 3130 XL semi-automated sequencer. For all accessions, both strands were sequenced and all sequences have been deposited in GenBank.
