# Supplementary material for: Cyto-nuclear discordance in the phylogeny of Ficus section Galoglychia and host shifts in plant-pollinator associations
Source: BMC Evol Biol. 2009 Oct 12;9:248. doi: 10.1186/1471-2148-9-248 (PMC2771017; doi:10.1186/1471-2148-9-248)
Supplement: Additional file 6 — List of species used for the development of non coding chloroplast DNA markers for Ficus. A table giving voucher numbers and origins. [file 1471-2148-9-248-S6.DOC]

**Additional file 6.** List of species used for the development of non coding chloroplast DNA markers for *Ficus.*

| **Section** | **Subsection** | **species** | **Sample code** | **Origin** | **Voucher** |
| --- | --- | --- | --- | --- | --- |
| *Pharmacosycea* |  | Ficus insipida Willd. | Fsp040*^2^* | French Guiana | conformity with Kjellberg 2006-006*^3^* |
| *Oreosycea* |  | *Ficus callosa* Willd. | Fsp031*^2^* | Thailand | - |
| *Americana* |  | *Ficus nymphaeifolia* P. Miller | Fsp004*^1, 2^* | Living collection (P) | Kjellberg 2008-12-25-04 |
|  |  | *Ficus guianensis* Desv. | Fsp039*^2^* | French Guiana | Kjellberg 2006-001 |
| *Conosycea* |  | Ficus benjamina L. | Fsp005*^1, 2^* | Living collection (P) | Kjellberg 2008-12-25-06 |
|  |  | *Ficus curtipes* Corner | Fsp033*^2^* | Thailand | - |
| *Malvanthera* |  | *Ficus macrophylla* Desf. | Fsp019*^2^* | Botanical garden, Lisboa | Lachaise 1994-04 |
| *Galoglychia* | *Caulocarpae* | *Ficus sansibarica sansibarica* Warburg | Fsp020*^2^* | Malawi | Lachaise 1991-05 |
|  |  | *Ficus bizanae* Hutch & Burt-Davy | Fsp025*^2^* | South Africa | Kjellberg 1999-27 |
|  |  | *Ficus polita* Vahl | Fsp027*^2^* | South Africa | Kjellberg 1999-03 |
|  | *Chlamydodorae* | *Ficus petersii* Warburg | Fsp024*^2^* | South Africa | Kjellberg 1999-06 |
|  |  | *Ficus craterostoma* Mildbraed & Burret | Fsp029*^2^* | South Africa | Kjellberg 1999-25 |
|  |  | *Ficus fisheri* Mildbraed & Burret | Fsp030*^2^* | South Africa | Kjellberg 1999-26 |
|  |  | *Ficus natalensis* Hochst. | Fsp035*^2^* | Uganda | Compton 2004-10-1-1 |
|  |  | *Ficus reflexa* Thunberg | Fsp036*^2^* | Madagascar | conformity with MNHN-Ma07-182*^3^* |
|  | *Cyatistipulae* | *Ficus tesselata* Warburg | Fsp011*^2^* | Ivory Coast | Lachaise 1994-01 |
|  |  | *Ficus lyrata* Warburg | Fsp018*^2^* | Ivory Coast | Lachaise 1993-03 |
|  |  | *Ficus scott-eliotti* Mildbraed & Burret | Fsp023*^2^* | Ivory Coast | Lachaise 1994-07 |
|  |  | *Ficus cyathistipula* Warb. | Fsp003*^1, 2^* | Living collection, unknown | Kjellberg 2008-12-25-01 |
|  | *Galoglychia* | *Ficus lutea Vahl* | Fsp001*^1, 2^* | Living collection (P) | Kjellberg 2008-12-25-02 |
|  |  | *Ficus saussureana* De Candolle | Fsp037*^2^* | Living collection (P) | Kjellberg 2008-12-25-03 |
|  | *Platyphyllae* | Ficus vasta Forssk. | Fsp002*^1, 2^* | Living collection (P) | Kjellberg 2008-12-25-05 |
|  |  | *Ficus stuhlmannii* Warburg | Fsp026*^2^* | South Africa | Kjellberg 1999-07 |
|  |  | *Ficus trichopoda* Baker | Fsp038*^2^* | Madagascar | - |
| *Urostigma* |  | *Ficus orthoneura* Léveillé & Vaniot | Fsp034*^2^* | China | Kjellberg 2006-07-22 |
| *Sycomorus* |  | *Ficus sur* Forrsk. | Fsp006*^1, 2^* | Living collection (P) | Kjellberg 2008-12-25-07 |
|  |  | *Ficus sycomorus* L. | Fsp008*^1, 2^* | live collection, Namibia | Compton 2000-09-11 |
|  |  | *Ficus tiliifolia* Baker | Fsp042*^2^* | Madagascar | Kjellberg 2007-523 |
| *Sycocarpus* |  | Ficus squamosa Roxb. | Fsp032*^2^* | Thailand | Kjellberg 2006-07-31 |
| *Ficus* |  | *Ficus palmata* Forrsk. | Fsp021*^2^* | Saudi Arabia | Lachaise 1994-06 |
|  |  | *Ficus carica* L. | Fsp009*^1, 2^* | Trabzon, Turkey | Kjellberg and Khadari 2002-TZ10 |
| *Sycidium* |  | *Ficus montana* Burm.f. | Fsp007*^1, 2^* | unknown | Kjellberg 2007-09-01 |
|  |  | *Ficus asperifolia* Miq. | Fsp041*^2^* | Uganda | Compton 2004-10-1-2 |

*^1^* First screening of cp consensus primers pairs [76]

*^2^* second screening of new cp primers defined in *Ficus.*

*^3^*Conformity verified by microsatellite markers
